# Potential of Anti-Cancer Activity of Secondary Metabolic Products from Marine Fungi

**DOI:** 10.3390/jof7060436

**Published:** 2021-05-30

**Authors:** Efaq Noman, Muhanna Mohammed Al-Shaibani, Muhammed Adnan Bakhrebah, Reyad Almoheer, Mohammed Al-Sahari, Adel Al-Gheethi, Radin Maya Saphira Radin Mohamed, Yaaser Qaeed Almulaiky, Wesam Hussain Abdulaal

**Affiliations:** 1 Department of Applied Microbiology, Faculty of Applied Science, Taiz University, Taiz 00967, Yemen; eanm1984@gmail.com; 2 Faculty of Applied Sciences and Technology, Universiti Tun Hussein Onn Malaysia (UTHM), Pagoh Higher Education Hub, KM 1, Jalan Panchor, Panchor 84000, Johor, Malaysia; 3 Micro-Pollutant Research Centre (MPRC), Faculty of Civil Engineering & Built Environment, Universiti Tun Hussein Onn Malaysia, Parit Raja, Batu Pahat 86400, Johor, Malaysia; muhanna@uthm.edu.my (M.M.A.-S.); mohammedalsahari@gmail.com (M.A.-S.); 4 Life Science and Environment Research Institute, King Abdulaziz City for Science and Technology (KACST), P.O. Box 6086, Riyadh 11442, Saudi Arabia; mbakhrbh@kacst.edu.sa; 5 School of Fundamental Science, Universiti Malaysia Terengganu, Kuala Nerus 21030, Terengganu, Malaysia; almohair4@yahoo.com; 6 Department of Chemistry, College of Science and Arts at Khulis, University of Jeddah, Jeddah 21921, Saudi Arabia; yasralmoliki@hotmail.com; 7 Chemistry Department, Faculty of Applied Science, Taiz University, Taiz 00967, Yemen; 8 King Fahd Medical Research Center, Cancer and Mutagenesis Unit, Department of Biochemistry, Faculty of Science, King Abdulaziz University, Jeddah 21589, Saudi Arabia; whabdulaal@kau.edu.sa

**Keywords:** anticancer, fungi, marine, L-asparaginase, production

## Abstract

The promising feature of the fungi from the marine environment as a source for anticancer agents belongs to the fungal ability to produce several compounds and enzymes which contribute effectively against the cancer cells growth. L-asparaginase acts by degrading the asparagine which is the main substance of cancer cells. Moreover, the compounds produced during the secondary metabolic process acts by changing the cell morphology and DNA fragmentation leading to apoptosis of the cancer cells. The current review has analyed the available information on the anticancer activity of the fungi based on the data extracted from the Scopus database. The systematic and bibliometric analysis revealed many of the properties available for the fungi to be the best candidate as a source of anticancer drugs. Doxorubicin, actinomycin, and flavonoids are among the primary chemical drug used for cancer treatment. In comparison, the most anticancer compounds producing fungi are *Aspergillus niger*, *A. fumigatus A. oryzae*, *A. flavus*, *A. versicolor*, *A. terreus*, *Penicillium citrinum*, *P. chrysogenum*, and *P. polonicum* and have been used for investigating the anticancer activity against the uterine cervix, pancreatic cancer, ovary, breast, colon, and colorectal cancer.

## 1. Introduction

**Figure 1:**
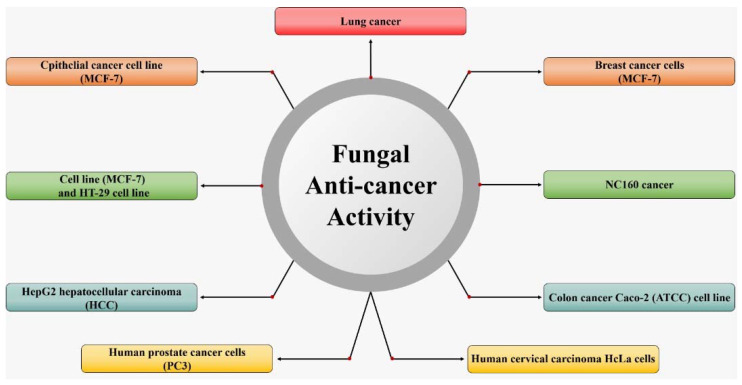
Applications of fungi in the environment to medicine.

Fungi have a variety of medical applications due to their capability of generating various enzymes and antimicrobial agents (Figure 1). It has been reported that fungi produce more antimicrobial and anticancer products than recorded by bacteria. However, these efficiencies and functions of the active compounds produced are dependent on the genes that cluster together in a genetic package, which are known as biosynthetic gene clusters (BGCs) [1]. Nonetheless, marine fungi represent a rich source of bioactive compounds and have yielded a wide range of anticancer compounds [2]. Kumar et al. [3] detected BGCs in *Calcarisporium* sp. and *Pestalotiopsis* sp. isolated from the German Wadden Sea.

The asparaginase enzyme which has been isolated from *A. niger* [4], *A. tubingensis* [5] *A. terreus* [6], *Fusarium* sp. [7], *Talaromyces pinophilus* [8], *Trichosporon asahii* [9], *Trichoderma viride* [10], and *Sarocladium strictum* [11] was recorded as the most anticancer properties among other several enzymes. Besides, several new anticancer products have been isolated and identified from the secondary metabolic production of marine fungi such as Gliotoxin which was isolated from *Aspergillus* sp. [12], Versicolactone B from *A. terreus* [13], Preussin from A. *candidus* [14], Patulin and Deoxytryptoquivaline from *A. giganteus* [15], and Octadecanoic from *Aspergillus* sp. [16]. Wijesekara et al. [17] purified physcion which induces cell apoptosis through down-regulating of Bcl-2 expression from *Microsporum* sp. They also reported that the compounds Nortryptoquivaline, 2,4-dihydroxy-3-methylacetophenone, chevalone C from *Neosartorya* siamensis exhibited strong effects against DNA and intracellular accumulation in lung cancer cells as well as induced cytotoxicity against the cancer cells and the cell death in lung cancer. The 3-hydroxy, benzenemethanol, 2-bromogentisyl alcohol, 2-chlorogentisyl alcohol, and Epoxydon 6-dehydroxy-6-bromogabosine C compounds purified from the secondary metabolic of *P. concentricum* exhibited anti-cancer activity against the human caucasian colon adenocarcinoma cells line [18]. The pentacyclic decalinoylspirotetramic acid and pyrenosetin D from *Pyrenochaetopsis* sp. showed anti-cancer activity against the melanoma cell line, the noncancerous keratinocyte cell line [19].

The current review aimed to highlight the applicability of marine fungi to produce anti-cancer compounds. A systematic literature review (SLR) methodology was performed in the current paper to avoid misinformation and to fill the gap in the literature. Furthermore, the bibliometric analysis of the anticancer literature in this article contributes strongly to creating a roadmap that leads the readers towards the right path to learning more information about the work.

## 2. Systematic Literature Review Methodology

**Figure 2:**
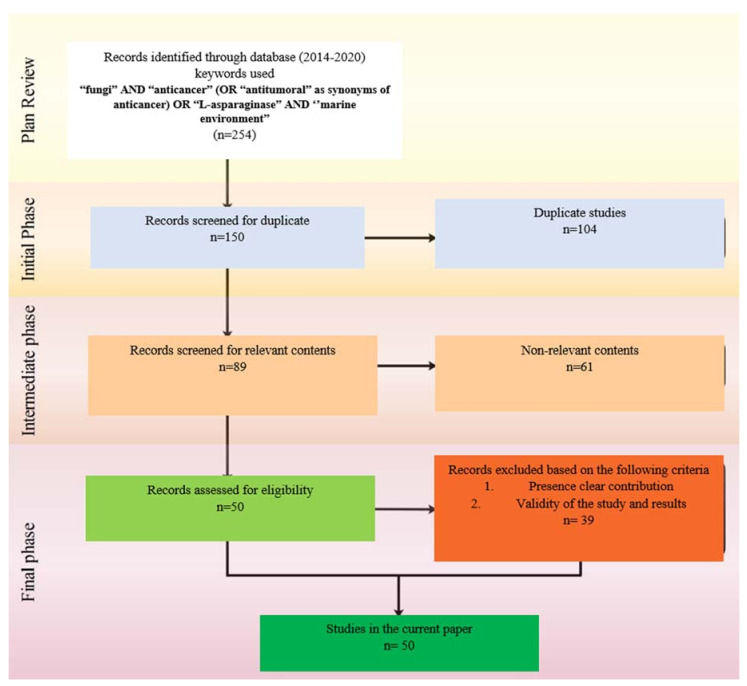
SLR Flowchart adopted with modification from the Preferred Reporting Items for Systematic Reviews and Meta-Analyses.

The systematic literature review methodology was performed in the current work according to the methods described in Figure 2 [20,21]. To classify the research points of interest and derive the vital results, two questions and objectives were stated (Table 1a). Table 1b shows the components of the current systemic review program.

The total number of final papers selected and used for reviewing the potential of secondary metabolic substances from marine fungi to be used as anticancer was 50, while 24 papers were used as references for basic concepts. The bias for the papers’ inclusion or exclusion threshold was performed according to Equation (1).

(1) $$R^{y}=\frac{\sum_{i}^{n}\frac{k_{i}}{n}}{P^{y}}\;$$

where R represents the papers’ relevance ratio of a particular year ( y), k represents the number of matches.

Keywords, n represent the total number of the proposed keywords, while P represent the number of the initial papers in a particular year. The included and excluded papers were determined according to Equation (2).

(2) $$f\left(k_{i}\right)\left\{\left\{\begin{array}{c} included,\;\;\;\;\;\;\;\;\;\;\;\;\;\;\;\;\;\;\;\;\;\;\;\;\;R^{y}<\frac{k_{i}}{P^{y}}\\ Excluded\;\;\;\;\;\;\;\;\;\;\;\;\;\left(otherwase\right) \end{array}\right.\right\}$$

Table 2 shows the included and excluded papers per publication year. According to the data analysis presented in Table 2, it can be noted that there was a continuous increase of publications during the last ten years, with 37.5% of the annual growth rate.

## 3. Bibliometric Analysis

**Figure 3:**
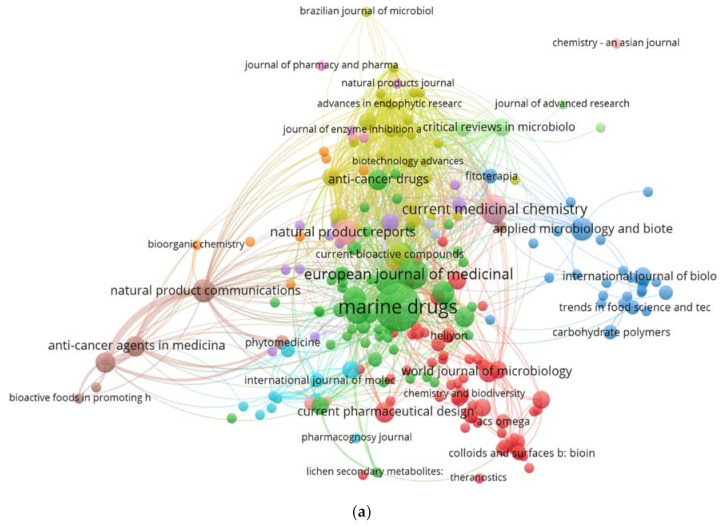

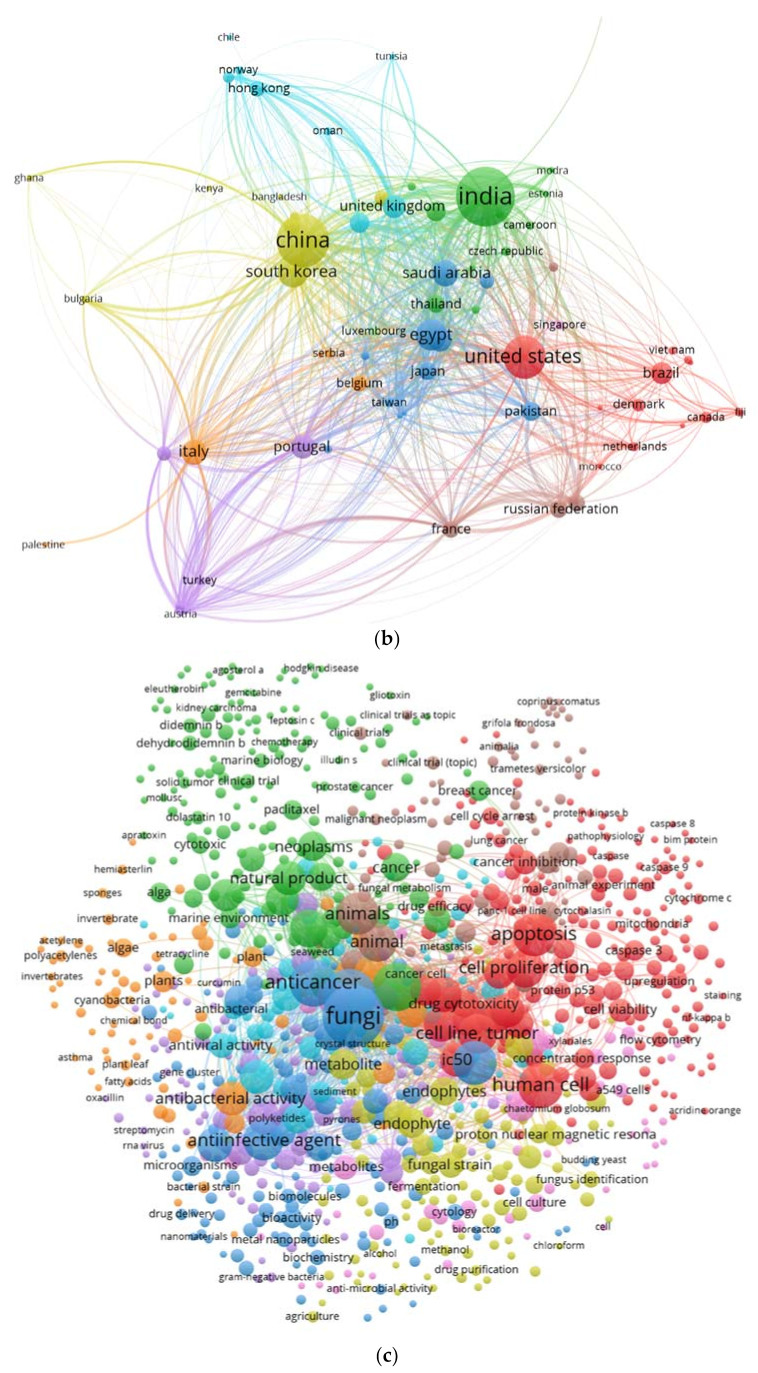
(**a**). Bibliometric analysis of the publication on the anticancer activity of fungi based on the journals. (**b**) Bibliometric analysis of the publication on the anticancer activity of fungi based on the countries. (**c**) Bibliometric analysis of the keywords of the anticancer activity of fungi papers.

The bibliometric analysis was used in the current research to assess the global trends of the fungal application in cancer treatment based on the Scopus literature database. The keywords used were “fungi” AND “anticancer” (OR “antitumoral” as synonyms of anticancer) OR “L-asparaginase” AND ‘’marine” the papers (254) extracted from the Scopus and PubMed database were selected between 2014 and 2020 and download as CSV file. The screening for the papers was conducted as described in the systematic process. In contrast, the VOSviewer software, Leiden University, The Netherland, Year (version 1.6.15) was used for the bibliographic data analysis based on the countries, journals, and keywords. All the obtained data of 254 documents, including 86 journals and 54 countries were imported to the VOSviewer software to be analyzed. In the journal productivity analysis, 79 journals (published 254 documents) were selected by the software for the provided data based on the TLS (high to low). The 30 most productive journals on anticancer from fungi were established. Marine Drugs, Journal of Photochemistry, Current Medicinal Chemistry, Frontiers in Microbiology, and the Natural Products Journal were the most common (Figure 3a). The color of the circles in the bibliographic map was used to assist in the definition of the journal′s section where similar keywords and contents of journals were linked closely together in the same area. The size of each circle expresses the strength of the journal based on the total publication (TP), total citation (TC), and total link strength (TLS).

According to the Scopus and PubMed databases, 30 countries have contributed to studying the potential of fungi to produce anticancer agents. India, China, USA, Korea, and Germany were at the top of the list indicating the critical role these countries play in the progress of biomedical drugs (Figure 3b).

Figure 3c show all elements which are used in anticancer research, the most common fungal used for the production of anticancer compounds are *Aspergillus niger*, *A.* wentii, *A. oryzae*, *A. terreus*, *A. flavus*, *A. versicolor*, *A. fumigatus*, *Penicillium citrinum*, *P. chrysogenum*, *P. polonicum*, as well as *Saccharomyces cerevisiae.* The products’ compounds from those organisms have been used for investigating the anticancer activity against uterine cervix cancer, pancreatic cancer, ovary cancer, breast cancer, colon cancer, colorectal cancer, hepatobiliary cancer, and lung cancer.

The main enzymes associated with cancer and anticancer include asparaginase, acyltransferase, α-glucosidase, α-amylase, β-glucosidase, β-glucuronidase, gelatinase, glutaminase, laccase, lactate dehydrogenase, triacylglycerol lipase, mono phenol mono-oxygenase, and xylan endo 1,3, β-xylosindase. Among several bacterial species that cause secondary infections associated with the cancer disease are Bacillus *cereus*, B. subtilis, Staphylococcus aureus, *Actinobacteria* sp., E. coli, Proteus vulgaris, *Pseudomonas aeruginosa* and *Streptococcus pyogenes*, while the most antibiotics used for the bacteria infection are gentamycin, pivalic acid, pivampicillin, and tetracycline. Furthermore, doxorubicin, actinomycin d, flavonoids, rapamycin, bleomycin, clidamycin, hyaluronic acid, mitomycin, and cisplatin are among the main chemical drugs used for cancer treatment.

## 4. Potential of Secondary Metabolic Substances from Marine Fungi as Anticancer Agents

The marine environment which includes the water, sediments, invertebrates, driftwood as well as marine mammals represents a rich source of fungi [18]. Up-to-date studies have estimated there are more than 10,000 marine fungal species, including the fungi belong to the Ascomycota and Basidiomycota phyla [22]. The marine environment has several secondary bioactive compounds of the marine natural products (MNPs) such as anticancer, antiviral, and antibiotic which are produced from the fungi. The organisms from the marine environment produce novel secondary metabolites (SMs). These secondary products have unique and diverse chemical structures as well as having high potential as novel drugs [23,24]. Hu et al. [25] reported that more than 2225 bioactive compounds from marine organisms exhibited anticancer activity. Polyphenols, polysaccharides, and alkaloids are among the highly active, biologically potent, and predominant anticancer compounds isolated from marine organisms as reported by [26,27].

According to the data analysis of reviewed papers and the summarising of the main findings, it was noted that the main enzymes associated with cancers are presented in Table 3. Sterol O-acyltransferase (ACAT1) enzyme is associated with increasing the cholesteryl ester levels. Therefore, in cancer treatment, the used drug acts by inhibiting the enzyme leading to suppression of proliferation in a variety of cancer cell types.

In contrast, α-amylase exhibited an anti-proliferative effect on breast cancer cells, while β-Glucosidase has an effective contribution in the inhibition of cancer cells, and the enzyme acts by combining with cancer-cell-surface antigens causing the conversion of amarogentin to an active drug acting on cancer cells. Some of the enzymes, such as Beta-glucuronidase (βG) are used as a biomarker for the diagnosis of cancer and prodrug therapy. In comparison, the laccases produced by basidiomycetes fungi have high potential as being anti-cancer as well as possessing anti-proliferative activities mainly against liver carcinoma cell lines and breast cancer. Understanding these enzymes and their role in cancer growth or inhibition represent the key element for cancer treatment, for instance, the inhibition or blocking of triglyceride lipases contributes effectively to prevent the development of cancer-associated cachexia [22,28].

**Figure 4:**
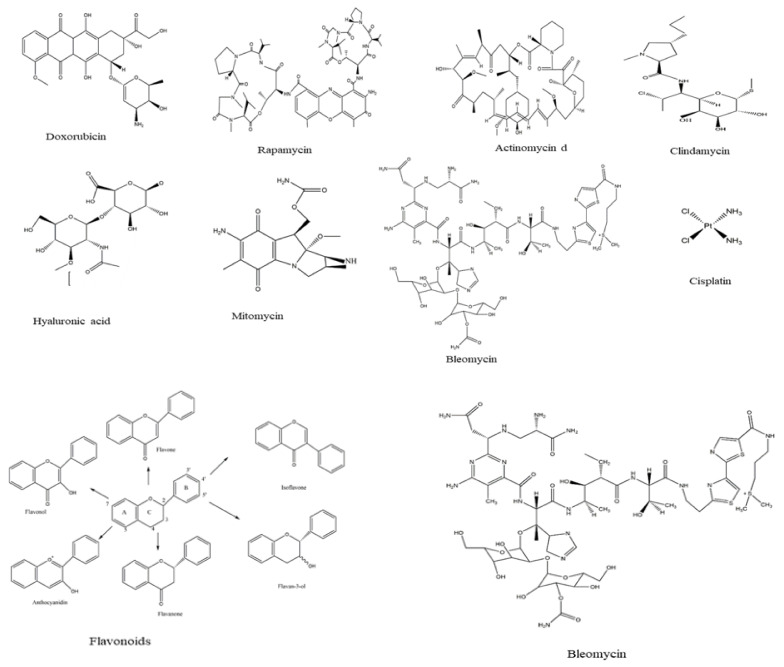
Chemical structure of drugs used for cancer treatment.

Some of the most common drugs used for cancer treatment and their side effects are illustrated in Table 4, the chemical structures are presented in Figure 4. Doxorubicin is the most common, recently used for treating hematological malignancies, soft tissue sarcomas, and acute lymphoblastic leukemia. The drug is approved by the Food and Drug Administration (FDA) and is among the most compelling of anticancer drugs. Nonetheless, clinical studies have revealed extreme restrictions of the drug, such as toxicity in normal cells and resistance. The main limitation lies in the negative consequences, including diarrhea, nausea, loss of appetite, vomiting, darkening of skin or nails, missed menstrual periods, tiredness, weakness, puffy eyelids, eye redness, as well as appearance of a reddish color to the urine [37]. According to Liu et al. [38], actinomycin is more effective against Wilms tumor, Ewing′s sarcoma, rhabdomyosarcoma, testicular cancer, trophoblastic neoplasia, and ovarian cancer, but the drug is associated with many side effects, especially low red and white blood cell levels. In contrast, Rapamycin is among a different type of anti-cancer which acts by inhibiting the tumor growth leading to halting tumor cell proliferation, and tumor cell apoptosis, and then suppressing tumor angiogenesis [39,40]. Hyaluronic acid has less toxicity as well as being biodegradable and non-immunogenic. Besides, the drug receptors are overexpressed on many tumor cells and have fewer side effects including pain, redness, bruising, swelling, and itching [41].

Several fungal species have been used as a source for the anti-cancer substances, among the fungi, the species that belong to *Aspergillus* spp. and Penicillium spp. have received great attention in the last few years, this is because these fungal species have the ability to produce several secondary metabolic products with anticancer properties against a variety of cancer cells, including caucasian colon adenocarcinoma cells, breast cancer cells, hepatocellular carcinoma, HeLa cells, pancreatic duct cancer, colorectal cancer cells, prostate cancer cells, and human chondrosarcoma cells.

The studies conducted on the possible fungal species in cancer treatment are illustrated in Table 5. The main compounds that have been detected in the secondary metabolic products and have recorded anti-cancer activity are 2,4-dihydroxy-3-methylacetophenone (1), Nortryptoquivaline (2), Chevalone C (3), Tryptoquivaline H (4), and Epifiscalin-C (5) which act by their effects on ultrastructural modifications, DNA damage, and intracellular accumulation in lung cancer cells, as well as obstructing cell proliferation, enhancing the intracellular accumulation of Dox, and triggering the cell’s death, and were isolated from *Neosartorya* spp. In addition, 2-Bromogentisyl alcohol (6) was isolated from *Penicillium* spp. Moreover, Patulin (8), Gliotoxin (10), Preussin (11), Deoxytryptoquivaline (12), Octadecanoic (13), and Versicolactone B (14) were isolated from *Aspergillus* spp (Table 5).

**Figure 5:**
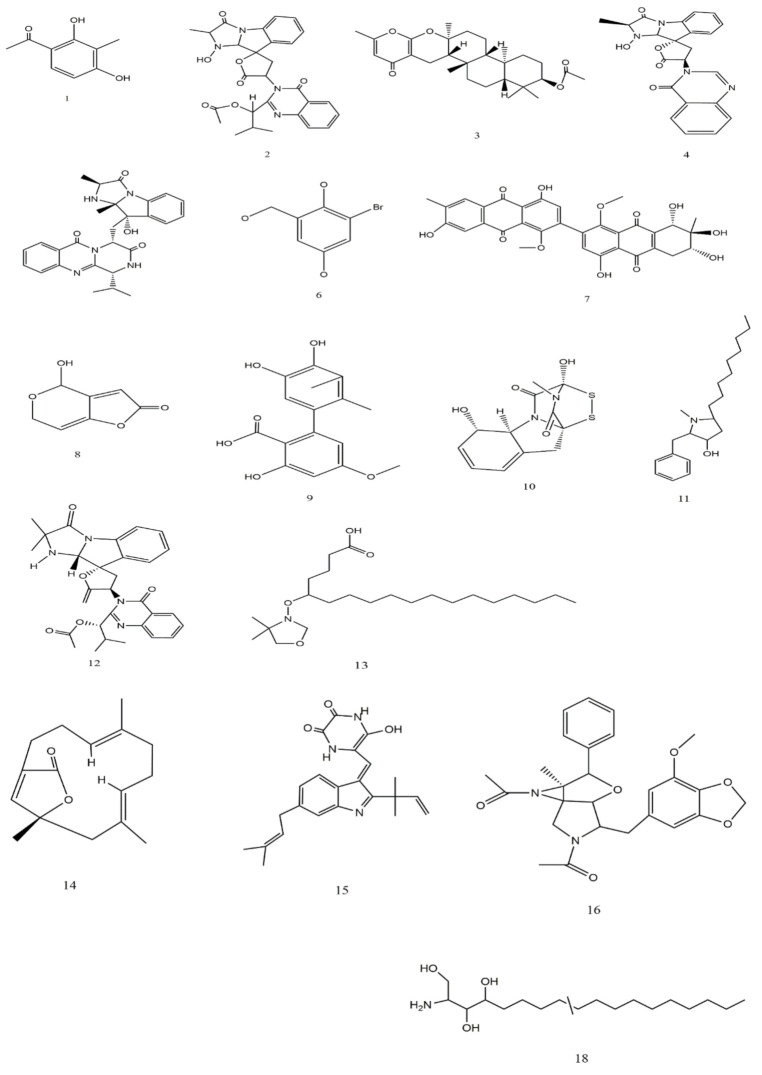

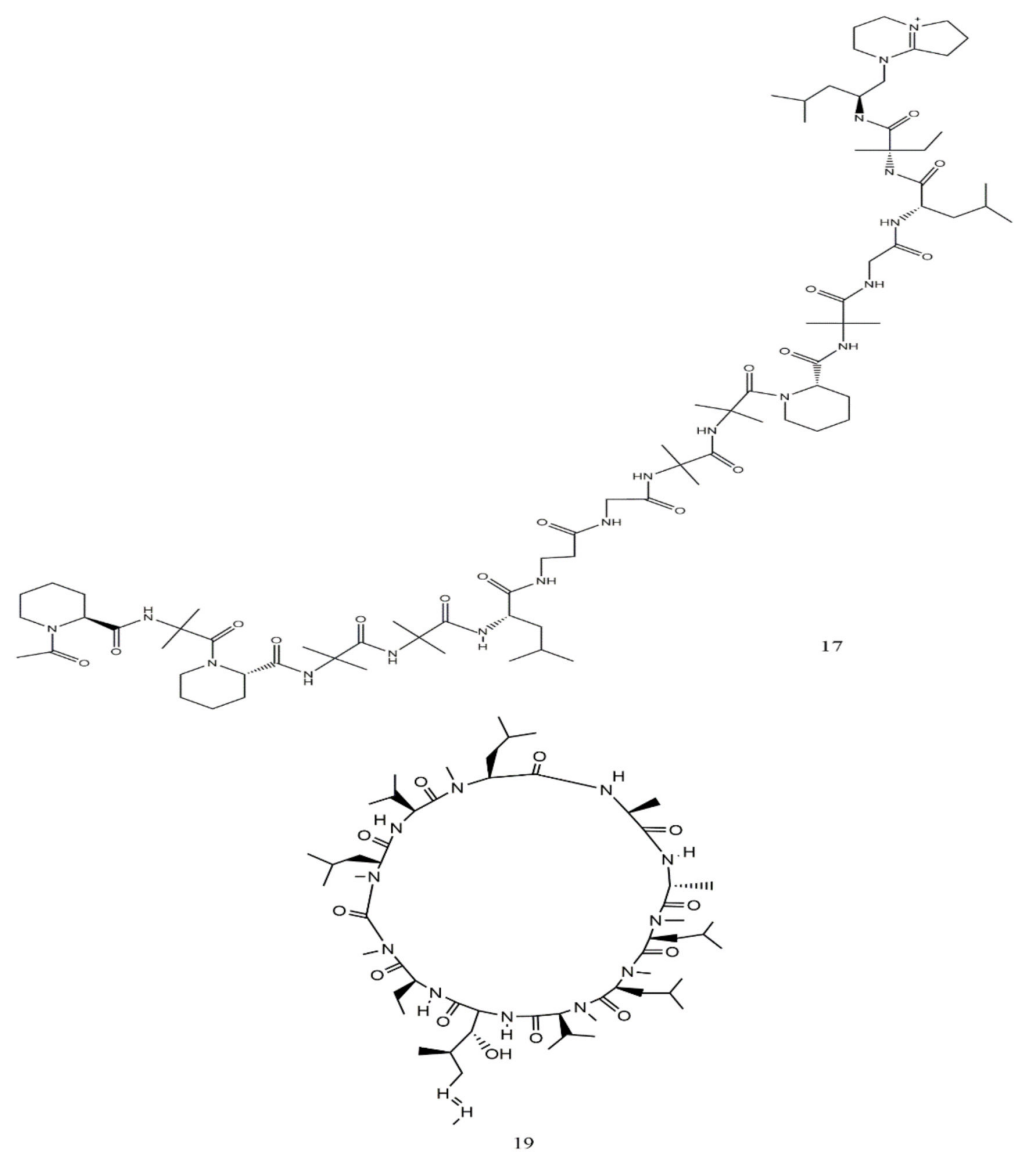
Chemical structure of some compounds from fungi with anti-cancer activity.

Alterporriol L (7), isolated from *Alternaria* sp., exhibited a high potential to change the cancer cell morphology and exhibit a significant inhibition for call growth, as well as inducing cancer cell apoptosis or necrosis in breast cancer cells lines [49]. Phthalide racemates (9), which has been isolated from the same fungal species exhibited cytotoxic activities against human erythroleukemia, human gastric carcinoma cells, and hepatocellular carcinoma cells [50]. Gliotoxin (10) (90 μM of concentration) from *Aspergillus* spp. has demonstrated anti-cancer activity and apoptosis of cancer cells and DNA fragmentation as well as induced activation of caspase-3, 8 and 9, down-regulation of Bcl-2, and up-regulation of Bax in human cervical cancer (Hela) and human chondrosarcoma cells [12]. The Butenolide derivatives, Asperlides A–C, Butenolides (+)-3′,3′-di-(dimethylallyl)-butyrolactone II and Versicolactone B (14) from *A. terreus* with IC_50_ values of 9.4 μM indicated anti-cancer activity against hepatocellular carcinoma, hepatocellular carcinoma, and pancreatic duct cancer [13]. Physcion from *Microsporum* sp. induced the cell apoptosis through down-regulating of Bcl-2 expression, up-regulating of Bax expression, as well as induced the formation of reactive oxygen species in HeLa cells [17]. The 2-Bromogentisyl alcohol, 3-hydroxy benzenemethanol, and Epoxydon compounds from *T. concentricum* have exhibited activity against prostate cancer cells and multiple cancer cells [51]. These compounds′ chemical properties are illustrated in Figure 5.

## 5. Application of L-Asparaginase as an Anti-Cancer Agent

**Figure 6:**
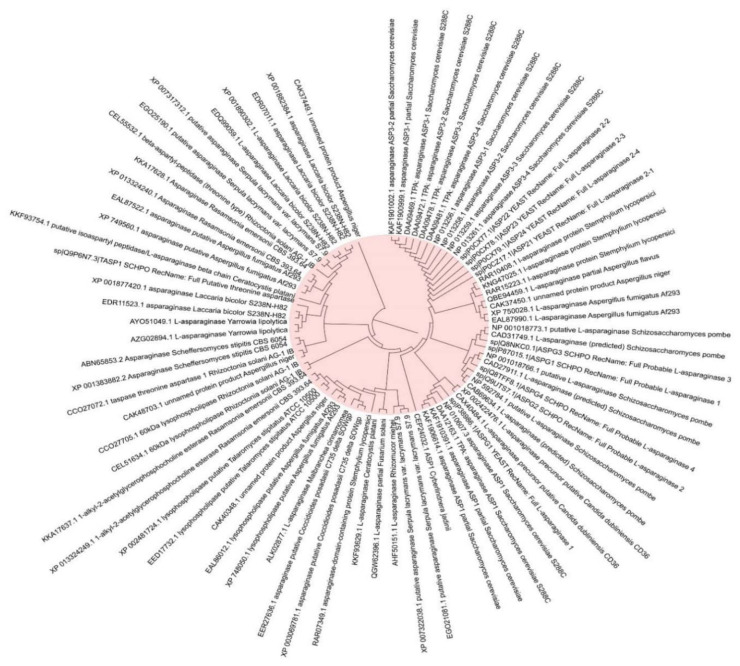
The most common fungal strains with high production of L-asparaginase.

L-asparaginase (ASNase) or L-asparagine amidohydrolase, (EC 3.5.1.1) is a hydrolase enzyme that has the ability to catalyze and hydrolyze L-asparagine into ammonia and L-aspartic acid. The enzyme has several clinical and medical applications for cancer treatment based on the differences between the metabolic pathway of the normal and cancer cells. The normal cells have the ability to synthesize L-asparagine while the cancer cells are totally dependent on the extracellular L-asparagine. Therefore, the enzyme acts by degrading the L-asparagine and leading to prevent the cancer cell growth caused by the lack of L-asparagine acquired for the development. The first study on the activity of L-asparaginase against cancer cells was reported in 1962, where the enzyme was tested against acute lymphoblastic leukemia. Several fungal species have been reported to produce ASNase among them *Fusarium oxysporum* (174 strains), *Fusarium fujikuroi* (90 strains), *Pyrenophora triticirepentis* (65 strains), *Aspergillus niger* (44 strains), *Alternaria tenuissima* (43 strains), *Aspergillus flavus* (40 strains), *Aspergillus fumigatus* (40 strains), *Penicillium expansum* (36 strains), *Fusarium graminearum* (33 strains), *Rhizoctonia solani* (33 strains), *Aspergillus oryzae* (30 strains), and *Trichophyton rubrum* (27 strains) (Figure 6) (The data were extracted from NCBI database as FASTA files and the plotted to Figure 6 using MEGAX, version 10.1.8).

Based on the summarized data presented in Table 6, the most common culture medium used for enzyme production is the Modified Czapek Dox (MCD). The fermentation process used in the production process is solid-state fermentation (SSF) and submerged fermentation (SmF), while several substrates have been used including wheat bran, cottonseed cake, and red gram husk, consecutive flaxseed oil cake (FOC), passion fruit peel flour, mustard oil cake (MOC), chicken viscera meal, sugarcane bagasse (SB), chicken feather meal, soybean, and rice meal [4,5,6]. In contrast, carbon source, sucrose, glucose, maltose, and lactose nitrogen source asparagine, glutamine, yeast extract, and peptone have been used as a carbon source [62], while urea, yeast extract, casein, malt extract, proline and peptone have been used as a nitrogen source [10]. In many of the experiments, the temperature was between 25 and 45 °C, time (1 to 7 days), agitation (0–250 rpm), and inoculum size ranged from 1–5 mL spores/100 mL, and pH between pH 5 to 8. The enzyme has exhibited stability at pH 4–10, temperature between 20 and 400 °C, with $K_{m}$ is 0.8141 mM and Vmax, 6.228 μM/mg/min [4]. Da Rocha et al. [63] noted that the enzyme was stable at pH 4–10, 20–40 °C, Tween 80 and Triton X-100 enhanced the activity, $K_{m}$ was 0.8141 mM, and Vmax was 6.228 μM/mg/min. The studies revealed that the maximum activity was between 20.58 to 84.3 U/gds after 120 h [5,6]. However, Grinde et al. [30] revealed that the highest activity recorded with passion fruit peel flour (2380.11 U/gds) after 48 h at 30 °C, 3746.78 U/gds with 60% of moisture and 2.1 × 10^6^ spores/g after 24 h at 25 °C.

The application of L-asparaginase is presented in Table 7. At a concentration of 125 μg/mL, L-Asparaginase showed high activity against lymphoma cancer cells (U937), with an IC_50_ value of 500 μg/mL of β-cyclodextrin-asparaginase nanobiocomposite [70]. In the anti-cancer studies of Baskar et al. [6], the L-Asparaginase is incorporated into the nano biocomposites which have been synthesized using β-cyclodextrin and chitosan to investigate the anti-cancer activity against prostate cancer cell lines and lymphoma cancer cells. The results revealed high activity against lymphoma cancer cells (U937) with IC_50_ value at 500 μg/mL of β-cyclodextrin-Asparaginase nanobiocomposite. Golbabaie et al. [11] used a crude L-Asparaginase enzyme mixed with cell viability to investigate the anti-cancer activity against K562 and HL60 cancer cell lines and lymphoblastic leukemia. The results revealed that the toxicity of the enzyme was determined with IC_50_ values were calculated as 0.4 and 0.5 IU/mL for K562 and HL60 respectively. Ertel et al. [71] used L-Asparaginase with a dose of ≥6000 IU/sq m three times weekly for treating childhood leukemia, the enzyme was effective in re-inducing remissions at 9.5% for 300 IU/sq m; 35.1% for 3000 IU/sq m; 53.5% for 6000 IU/sq m; and 62.5% for 12,000 IU/sq m.

## 6. Conclusions

This review has attempted to cover the aspects for anticancer agents from marine fungi, the reader can get an idea about the different fungal species which have been investigated as a source for the compounds used for the cancer treatment. The marine environment represents a rich source of fungi that has the ability to produce several secondary bioactive compounds from the MNPs such as anti-cancer, antiviral and antibiotic which are produced from these fungi. Moreover, more than 2225 bioactive compounds from marine organisms exhibited anti-cancer activity. Some of the most biologically potent and predominant anti-cancer compounds have been isolated from marine organisms, these secondary products have unique and diverse chemical structures as well as has high potential novel drugs such as polyphenols, polysaccharides, and alkaloids. The main enzymes associated with cancer and anticancer include asparaginase, acyltransferase, α-glucosidase, α-amylase, β-glucosidase, β-glucuronidase, gelatinase, glutaminase, laccase, lactate dehydrogenase, triacylglycerol lipase, mono phenol mono-oxygenase, and xylan endo 1,3, β-xylosindase. The mode of action of these drugs acts by inhibiting the enzyme leading to suppression of proliferation in a variety of cancer cell types. The following findings by various *researcher’s* point to the possibility of anti-cancer agents from fungi: the anti-cancer agents from fungi act by cytotoxic activities and apoptosis of cancer cells and DNA fragmentation as well as by hindering cell proliferation, enhancing Dox aggregation intracellularly, and causing the cell’s death.

## Figures and Tables

**Table 1:** (**a**) Systematic Literature Review (SLR) objectives and questions. (**b**) Research plan of the SLR.

(a)		
No.	Objectives	Questions
1	To identify the potential application of secondary metabolic substances as anti-cancer against	How do secondary metabolic substances affect cancer cells?
2	To verify the effectiveness of the asparaginase enzyme against cancer cells	How does the asparaginase work to inhibit the cancer cells?
**(b)**		
**Categories**	**Description**	
Context	This SLR presents the potential of secondary metabolic substances as anti-cancer.	
Objectives	The objective of the systematic review is to answer the questions regarding the application of secondary metabolic substances for cancer treatment.	
Method	The method used in this SLR process is data collection, screening, data verification, data analysis, and discussion.	
Result	The result shows the performance of each advanced technology, the advantages, and disadvantages of each method.	
Conclusion	The discussion regarding the objectives and questions is successfully achieved with clear discussion.	

**Table 2:** The included and excluded relevant papers per publication year.

Year	Initial	R	Included	Excluded
2020	22	0.0182	4	18
2019	36	0.0111	5	31
2018	29	0.0207	3	26
2017	28	0.0143	4	24
2016	22	0.0364	5	17
2015	24	0.0167	3	21
2014	27	0.0222	7	20
2013	17	0.0471	6	11
2012	20	0.0500	4	16
2011	13	0.0462	3	10
2010	16	0.0625	6	10
Total	254	0.3452	50	204

**Table 3:** Most common enzymes associated with cancer in humans.

Enzyme	Molecular Structure	Gene Location (Human)	Role	References
Sterol O-acyltransferase (ACAT1)	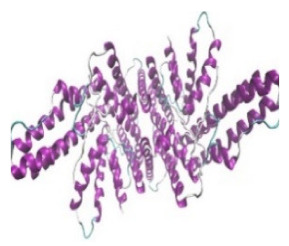	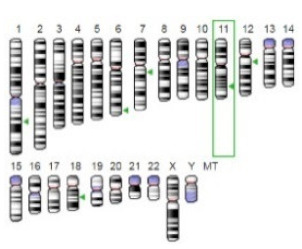	The enzyme is involved in a variety of cancer types because of its association with increasing cholesteryl ester levels. The anti-cancer drug acts by inhibiting the enzyme leading to suppression of proliferation in a range of cancer cell types.	[29]
Glutaminase	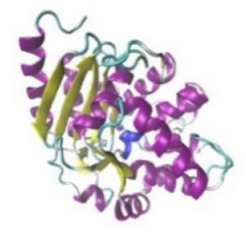	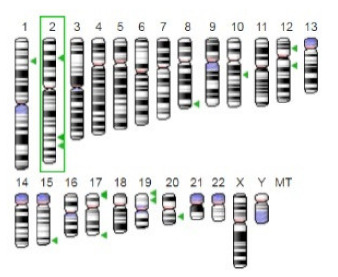	The enzyme acts by inhibiting the target breast cancer cells by blocking the conversion of glutamine to glutamate. Therefore, some studies indicated that the enzyme is thereby potentially interfering with anaplerosis and the synthesis of amino acids and glutathione.	[30]
β-Glucosidase	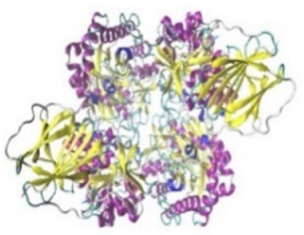	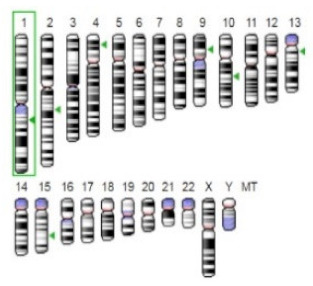	β-Glucosidase plays an essential role in the inhibition of cancer cells by combing with cancer-cell-surface antigens leading to converting amarogentin to an active drug that acts on cancer cells and the surrounding antibodies to achieve a killing effect.	[31]
Lactate dehydrogenase B (LDHB)	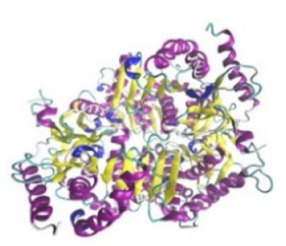	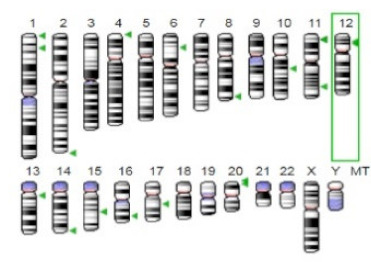	The enzyme is an intracellular enzyme and is released into the bloodstream when the cells are damaged due to tissue destruction caused by tumor growth. Therefore, the increase of enzyme levels in the blood is usually used as indicators of tissue damage.	[32]
Laccase	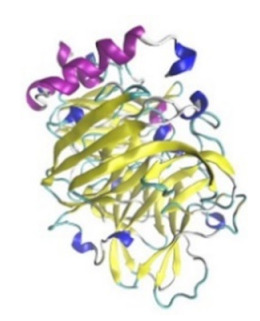	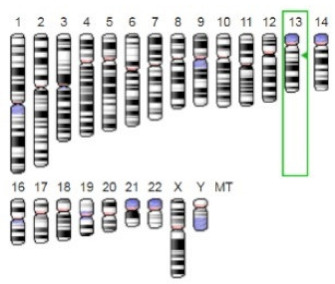	Laccases from basidiomycetes fungi exhibited high potential as anti-cancer as well as having anti-proliferative activities primarily against breast cancer and liver carcinoma cell lines.	[33]
β-Glucuronidase	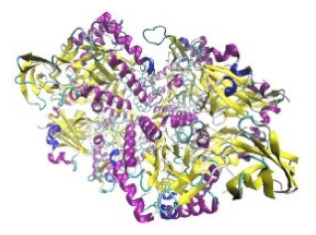	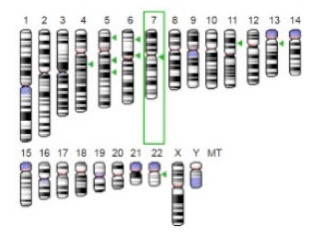	Beta-glucuronidase (βG) is a biomarker for the diagnosis of cancer and prodrug therapy. Therefore, the image βG activity in patients is associated with the personalized glucuronide prodrug cancer therapy.	[34]
α-amylase	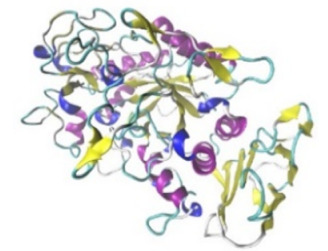	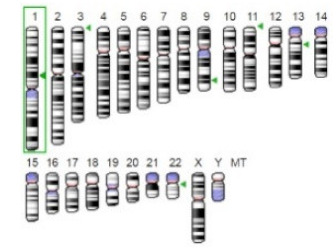	The experiments conducted on the primary cell cultures of human breast cancer cells exhibited an anti-proliferative effect for salivary α-amylase.	[35]
Matrix metallopeptidase 9 (MMP-9)	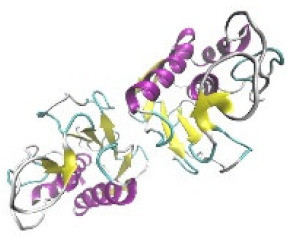	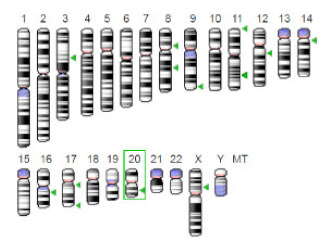	The modulation of gelatinase expression in the host cells is associated with the interactions between cancer cells and host tissues. Therefore, the inhibition of gelatinases by synthetic MMP inhibitors is an attractive approach to block cancer progression.	[36]

**Table 4:** Some common drugs used for cancer treatment and their side effects.

Drug	Formula	Utilization	Side Effects	References
Doxorubicin	C_17_H_30_ClNO_11_	The drug is used as anti-cancer for a wide range of cancers such as hematological malignancies, soft tissue sarcomas, and acute lymphoblastic leukemia	Vomiting, nausea, loss of appetite, diarrhea, darkening of skin or nails, missed menstrual periods, tiredness, weakness, puffy eyelids, eye redness, as well as the appearance of a reddish color to urine, tears, and sweat.	[42]
Actinomycin d	C_62_H_86_N_12_O_16_	The drug has anti-cancer properties against Wilms tumor, Ewing′s sarcoma, rhabdomyosarcoma, testicular cancer, trophoblastic neoplasia, and ovarian cancer.	The drug is associated with low red and white blood cell levels, decrease in the low platelet levels leading to increased risk of infection, anemia, and bleeding. Nausea and vomiting, hair loss, sores in the mouth, skin reactions, diarrhea, acne, peeling, skin eruptions, and sensitivity to sunlight.	[43]
Flavonoids		The flavonoids have high potential as anti-cancer agents and exhibited great potential against cancer cells.	The side effects have been reported for the flavonoids. However, some reports indicate the presence of anemia, fever, and hives which have been disappeared when treatment was discontinued.	[44]
Rapamycin	C_51_H_79_NO_13_	The drug acts by inhibiting the tumor growth leading to halting tumor cell proliferation, and tumor cell apoptosis, and then suppressing tumor angiogenesis.	The side effects of the drug include stomatitis and mycositis which are associated with high doses or long term used. Moreover, some studies reported non-infectious interstitial pneumonitis and hyperglycemia.	[40]
Clindamycin	C_18_H_33_ClN_2_O_5_S	Clidamycin which is classified as a member of the enediyne anti-cancer antibiotic family exhibited cytotoxicities against cancers in vitro and in vivo.	Nausea, diarrhea, and vomiting, heartburn, metallic taste in the mouth, abdominal and joint pain, skin rash, redness, itching, vaginal itching, and burning.	[45]
Hyaluronic acid	(C_14_H_21_NO_11_)_n_	The drug is common because it is biocompatible, non-toxic, biodegradable, and non-immunogenic, as well as HA receptors are overexpressed on many tumor cells	Pain, redness, bruising, swelling, and itching.	[41]
Mitomycin	C_15_H_18_N_4_O_5_	Mitomycin is anti-cancer produced by *Streptomyces caespitosus* and exhibited high efficiency against a wide variety of cancer types	Anorexia, fever, vomiting, and nausea, as well as a blurring of vision, headache, drowsiness, confusion, fatigue, syncope, thrombophlebitis, anemia, diarrhea, hematemesis, and pain	[46]
Cisplatin	[Pt(NH_3_)_2_Cl_2_]	The drug is among the most effective anti-cancer against solid tumors and acts by damaging DNA and inhibiting DNA synthesis.	Nausea, low blood counts, vomiting, ototoxicity hearing loss, ringing in the ears, kidney toxicity, blood test abnormalities	[47]
Bleomycin	C_55_H_84_N_17_O_21_S_3_	The drug is used in combination with surgery or radiotherapy against squamous cell cancers, sarcoma, melanoma, both Hodgkin′s and non-Hodgkin′s lymphoma as well as testicular cancer	Fever, chills, redness, stretch marks, and darkening of the skin, peeling and thickening, nail thickening and banding as well as hair loss.	[48]
Doxorubicin	C_17_H_30_ClNO_11_	The drug is used as anti-cancer for a wide range of cancers such as hematological malignancies, soft tissue sarcomas, and acute lymphoblastic leukemia	Vomiting, nausea, loss of appetite, diarrhea, darkening of skin or nails, missed menstrual periods, tiredness, weakness, puffy eyelids, eye redness, as well as the appearance of a reddish color to urine, tears, and sweat	[42]
Actinomycin d	C_62_H_86_N_12_O_16_	The drug has anti-cancer properties against Wilms tumor, Ewing′s sarcoma, rhabdomyosarcoma, testicular cancer, trophoblastic neoplasia, and ovarian cancer.	The drug is associated with low red and white blood cell levels, decrease in the low platelet levels leading to increased risk of infection, anemia, and bleeding. Nausea and vomiting, hair loss, sores in the mouth, skin reactions, diarrhea, acne, peeling, skin eruptions, and sensitivity to sunlight	[43]
Flavonoids		The flavonoids have high potential as anti-cancer agents and exhibited great potential against cancer cells.	Side effects reported for the flavonoids indicate the presence of anaemia, fever, and hives which have been disappeared when treatment was discontinued	[44]
Rapamycin	C_51_H_79_NO_13_	The drug acts by inhibiting the tumor growth leading to halting tumor cell proliferation, and tumor cell apoptosis, and then suppressing tumor angiogenesis.	The side effect of the drug includes stomatitis and mycositis which is associated with high doses or chronically used. Moreover, some studies reported non-Infectious interstitial pneumonitis and hyperglycemia	[40]
Clindamycin	C_18_H_33_ClN_2_O_5_S	Clidamycin which is classified as a member of the enediyne anti-cancer antibiotic family exhibited cytotoxicities against cancers in vitro and in vivo.	Nausea, diarrhea, and vomiting, heartburn, metallic taste in the mouth, abdominal and joint pain, skin rash, redness, itching, vaginal itching and burning.	[45]
Hyaluronic acid	(C_14_H_21_NO_11_)_n_	The drug is common because it is biocompatible, non-toxic, biodegradable, and non-immunogenic, as well as the HA receptors are overexpressed on many tumour cells	Pain, redness, bruising, swelling, and itching	[41]
Mitomycin	C_15_H_18_N_4_O_5_	Mitomycin is anti-cancer produced by Streptomyces caespitosus and exhibited high efficiency against a wide variety of cancer types	Anorexia, fever, vomiting, and nausea, as well as a blurring of vision, headache, drowsiness, confusion, fatigue, syncope, thrombophlebitis, anemia, diarrhea, hematemesis, and pain	[46]
Cisplatin	[Pt(NH_3_)_2_Cl_2_]	The drug is among the most effective anti-cancer against solid tumours and acts by damaging DNA and inhibiting DNA synthesis.	Nausea, low blood counts, vomiting, ototoxicity hearing loss, ringing in the ears, kidney toxicity, blood test abnormalities	[47]

**Table 5:** Review of the studies conducted on the application of secondary metabolic products from fungi as anti-cancer agents.

Fungal Genus	Fungal Species	Active Substances	Effectiveness against Cancer	References
*Alternaria*	*Alternaria* sp. obtained from the fruit of a mangrove tree *Aegiceras corniculatum*	Alterporriol L (7)	Alterporriol L changed the cancer cell morphology and exhibited a significant inhibition of cell growth, as well as inducing cancer cell apoptosis or necrosis in breast cancer cells lines.	[49]
	*Alternaria* sp. from a *Callyspongia* sp. sponge	Perylenequinone derivatives An altenusin derivative Phthalide racemates (9), Phenol derivatives.	These compounds exhibited cytotoxic activities against human erythroleukemia, human gastric carcinoma cells, and hepatocellular carcinoma cells.	[50]
*Aspergillus*	*Aspergillus* sp., from marine brown algae	Gliotoxin (10)	Anti-cancer activity and apoptosis of cancer cells and DNA fragmentation, as well as induced activation of caspase-3, 8 and 9, down-regulation of Bcl-2, up-regulation of Bax in human cervical cancer (Hela) and human chondrosarcoma cells.	[12]
	A. *candidus* associated with the marine sponge *Epipolasis* sp.	Preussin (11) (10 µM)	Preussin exhibited an ability to cause cell death as confirmed by caspase-3 immunostaining of breast cancer cells.	[14]
	*Aspergillus giganteus* isolated from Ulva lactuca	Aspergilsmins A–G, Patulin (8), Deoxytryptoquivaline (12), tryptoquivaline Quinadoline B. IC_50_ values between 2.7–7.3 µM	The compounds have exhibited anti-cancer activity against human hepatocellular carcinoma cells and prostate cancer cells.	[15]
	*Aspergillus* sp.	Hexadecanoic, Octadecanoic (13), Octadecenoic acids.	The compounds had significantly high cytotoxic activity against colorectal cancer cells.	[16]
	*A.Protuberus* isolated from marine sediments	*n*-butanol extract of mycelium	The extract exhibited anti-cancer activity against the Hep 2 cell line.	[52]
	*A. terreus* from sea deposit	Butenolide derivatives, Asperlides A–C Butenolides (+)-3′,3′-di-(dimethylallyl)-butyrolactone II, Versicolactone B (14)	The compounds exhibited anti-cancer activity against hepatocellular carcinoma, hepatocellular carcinoma, and pancreatic duct cancer.	[13]
*Aspergillus* *Neosartorya* *Talaromyces*	*A. similanensis*, *N. paulistensis*, *and T. trachyspermus*	Crude ethyl acetate extracts	The extract exhibited anti-cancer activity against HepG2, HCT116, and A375.	[51]
*Microsporum*	*Microsporum* sp. isolated from the surface of a marine red alga *Lomentaria catenata*	Physcion (11.8 mg)	The compound induces cell apoptosis through down-regulating of Bcl-2 expression, up-regulating of Bax expression, as well as induced the formation of reactive oxygen species in HeLa cells.	[17]
	*Microsporum* sp. isolated from the surface of marine red algae, *Lomentaria catenata*,	Physcion physcion activated caspase-3,8, 9, Ras, Bcl-xL, and Bcl-2 Bax (0–50 µM)	Physcion decreases cell proliferation and induces cell apoptosis in human prostate cancer cells.	[53]
*Neosartorya*	*N. pseudofischeri*	1,4-diacetyl-2,5-dibenzylpiperazine (16), Derivative, A quinazolinone-containing indole derivative, A new ester of 2,4-dihydroxy-6-methylbenzoic acid.	The compounds exhibited anti-cancer activity against human glioblastoma and non-small cell lung cancer Apoptosis-resistant cells, and distinct cancer cell lines.	[54]
	*N. siamensis* isolated from *Rumphella* sp. sea fan	2,4-dihydroxy-3-methylacetophenone (1), Nortryptoquivaline (2) Chevalone C (3), tryptoquivaline H (4), Epifiscalin-C (5) (0.54 to 100 µg/mL)	Effects on DNA damage, ultrastructural modifications, and intracellular accumulation in lung cancer cells. NS extract has cytotoxicity by inhibiting cell proliferation, increasing intracellular accumulation of Dox, and inducing cell death in lung cancer	[51]
*Paradendryphiella*	*P. salina* (from the brown alga *Pelvetia caniculata*)	Hyalodendrin (251.53 mg)	The compound induces the changes in the phosphorylation status of p53 and altered expression of epithelial cancer cell line	[55]
*Penicillium*	*P. concentricum* isolated from the healthy liverwort Trichocolea tomentella	2-Bromogentisyl alcohol (6), 3-hydroxy, benzenemethanol, Epoxydon 6-dehydroxy-6-bromogabosine C, 2-chlorogentisyl alcohol, gentisyl alcohol Griseofulvin (IC_50_ values of 8.4, 9.7, and 5.7 μM)	The compounds exhibited anti-cancer activity against the human caucasian colon adenocarcinoma cells line	[18]
	*Penicillium* sp. isolated from marine sediments	(Z)-Octadec-9-enamide (oleamide) IC_50_ = 22.79 μg/mL	Anti-cancer activity against breast cancer cells.	[56]
	*P. citrinum* from marine sediments	Penicitrinine A (12–100 µM)	The compound induces A-375 cell apoptosis by decreasing the expression of Bcl-2 and increasing the expression of Bax in multiple tumor types	[38]
	*P. oxalicum* (from marine algae *Chaetomorpho anteria*)	Anthraquinone Cinnamic acid. (20–100 µg/mL)	The extract enhanced the membrane damage and apoptosis in breast cancer cells. The extract inhibited anti-cancer activity against human breast cancer and HeLa cells.	[57]
*Penicillium, Cladosporium, Emericellopsis and Plectosphaerella*	*Penicillium, Cladosporium, Emericellopsis and Plectosphaerella*	Crude extracts	The extracts exhibited inhibitory potential anti-cancer against A-549 lung carcinoma cells, breast cancer, and human keratinocytes.	[58]
*Phoma*	*Phoma* sp. (obtained from the marine sponge *Ectyplasia perox*)	Epoxyphomalin A and B (IC_50_, 0.17–0.33 μg/mL]	The compound has strong cytotoxic properties in different cancer cell lines.	[59]
*Pyrenochaetopsis*	*Pyrenochaetopsis* sp. -from *Fucus vesiculosus*	Pentacyclic decalinoylspirotetramic acid, Pyrenosetin D (IC_50_ values of 77.5 and 39.3 µM)	Pyrenosetin D showed anti-cancer activity against the melanoma cell line and noncancerous keratinocyte cell line.	[7]
*Talaromyces*	*T. flavus* SP5 from marine sediment	Gusation A, 2-amino-1,3,4-trihydroxy-8-octadecene (18), Vitamin E, Tetradecanoic acid, 12-dimethyl-methyl ester, 2-*O*-benzyl-3,4-*O* isopropylidene-D-rythrosedi-ethyldithioacetal, methyl, acetate, 2,5-bis (4-methoxyphenyl)thiophene, and chalcone(LC50 value of 25.7 μg/mL)	The compounds exhibited anti-cancer activity against the HEp2 carcinoma cell line.	[60]
*Tolypocladium*	*T. geodes* isolated from a sponge sample.	Cyclosporin A (19), Efrapeptin D (17), Pyridoxatin, Terricolin A, Malettinins B and E, Tolypocladenols (different IC_50_)	Anti-tumor effects against cancer cell line panel.	[61]

**Table 6:** Experimental conditions for producing L-asparaginase from different fungal species.

Fungal Genus	Fungal Species	Production/Medium/ and Substrate	Factors Investigated	Characteristics	References
*Aspergillus*	*A. niger* AKV-MKBU	MCD	Tween 80 and Triton X-100, pH 4–10	Stable at pH 4–10, at 20–400 °C. $K_{m}$ is 0.8141 mM and V_max,_ 6.228 μM/mg/min.	[4]
	*A. tubingensis* IBBL1	SSF (Tray bioreactor) Cottonseed cake, wheat bran, and red gram husk consecutive	pH-8, 2%(*w/w*) inoculum, room temperature	Maximum activity was 20.58 U/gds after 120 h	[5]
	*A. terreus*	SmF and SSF, flaxseed oil cake, mustard oil cake, sugarcane bagasse	Temperature (25–45 °C), time (96–192 h), agitation (0–250 rpm) and inoculum (1–5 mL/100 mL media), l-asparagine and dextrose, pH 5.5.	The highest activity (84.3 U) with flaxseed oil cake as the substrate for the production of a purified enzyme with 6.39- fold purity.	[6]
	*A. terreus*	MCD	pH 6.2, 120 rpm, 32 °C, 72 h	The activity was 47.29 U/ mL.	[6]
	*A. niger* LBA 02	SSF wheat bran, soybean meal, rice meal, chicken feather meal, chicken viscera meal, passion fruit peel flour	Temperature (25–35 °C), initial moisture content (40–60%) and inoculum concentration (2.1–7.99 × 10^6^ spores/g).	The highest activity recorded with passion fruit peel flour (2380.11 U/gds) after 48 h at 30 °C, 3746.78 U/gds with 60% of moisture and (2.1 × 10^6^) spores/g after 24 h at 25 °C.	[64]
	*A.* *tamarii*	SmF MCD	Incubation periods (1, 2, 3, 4, 5, 6, 7, 8, and 9 days), temperatures (25, 30, 35, and 40 °C). pH (2, 3, 4, 5, 6, 7), carbon source, sucrose, glucose, maltose, and lactose nitrogen source asparagine, yeast extract, peptone, and glutamine.	The highest activity (11.01 u/mL) at 30 °C for 7days of incubation pH 7.0, sucrose is the most effective carbon source, with L-asparagine as sole nitrogen source	[59]
	*A. niger* SVUAn1	MCD	30 °C and incubation periods (24–144 h), pH 6.2, 0.2% of carboxy-methylcellulose, fructose, maltose, starch, sucrose, and yeast extract.	Maximum production achieved at 96 h (4 days) with the incorporation of glucose as a carbon source in the culture medium	[65]
	*A. terreus*	Fermentation in a 5-l bioreactor system with MCD medium	30 °C, 120 rpm, 96 h, glucose, sucrose, lactose or fructose, arginine, glutamine, asparagine, tyrosine, leucine, tryptophan, or histidine), urea, ammonium nitrate, sulfate.	Maximum production is of 108 U with glucose, proline, and asparagine.	[63]
*Fusarium*	*Fusarium* sp. LCJ273	SmF MCD Broth and wheat bran	Dextrose, ammonium sulfate Production at 120 rpm for 5–8 days.	Activity was recorded 9.18 ± 0.9 U/mL, at 3 g/L Dextrose, 20 g/L ammonium sulphate and 13.69 ± 0.4 U/mL at 2.5 g/L wheat bran after 5 d.	[7]
*Penicillium* sp. *Fusarium* sp.	*Penicillium* sp. T6.2) (*Fusarium* sp.)	stationary liquid state bioprocesses Medium Bacelar-1 Medium	Glycerol; L-asparagine; 10^5^ mL^−1^ of inoculum, incubated for 72 h at 30 °C.	The enzyme activity was 8.3 U min/ mL from *Penicillium* sp.) and 11.4 U min/ mL from *Fusarium* sp. after 72 h in Bacelar-1 medium.	[66]
*Penicillium*	*Penicillium* sp.	the fermentation process in potato dextrose broth wheat bran	Temperature (25–35 °C), the incubation period (48–96 h), and initial pH (5–9), L-proline, L-asparagine, ammonium sulfate, yeast extract, sucrose, and glucose.	Maximum activity (2.33 IU/mL) detected at 2.8% L-asparagine 4.0% L-proline, 0.75% Potato dextrose broth, and 0.1% Sucrose.	[67]
	*Penicillium* sp. LAMAI 505	SSF multiple reactors with immobilized cells	pH level (pH), residence time (RT), the time between cycles (TC), and concentration of glucose and L-asparagine.	L-asparaginase activity was 13.7 U/gds was achieved at a residence time of 33.5 min, pH of 5.1, and concentrations of L-asparagine and glucose of 1.2 and 3.0 g/L.	[68]
*Talaromyces*	*T. pinophilus*	MCD	Agitation rate (100–150 rpm), pH (4.0–9.5), temperature (15–40 °C), and (7–29 days), glucose (2–15), starch (2.5–15), yeast extract (2.5–15), and L-asparagine (5–15).	The enzyme activity of 108.95 U/mL was recorded at 120 rpm after at 120 h with L-asparagine, and starch as the carbon source than glucose.	[69]
*Trichosporon*	*T. asahii* IBBLA1	NA	Temperature, pH, L-Asparagine concentration and glucose concentration.	Optimum enzyme activity of 20.57 U mL^−1^ was obtained at 30 °C and pH of 7.0 after 60 h	[9]
*Trichoderma*	*T. viride* F2	SSF maize, rice bran, rice husk, wheat bran, wheat germ, rice straw, cottonseed wastes	pH value (3.0–8.0), 50 to 86% of moisture content, incubation temperature (25, 28, 35, and 40 °C). Inoculum size (1 × 10^4^–1 × 10^9^), surfactants (Tween 20,60 and 80 and Triton X-100 at 0.1% *w/v*). Carbon source (Glucose, sucrose, maltose, fructose, xylose, galactose, arabinose, soluble starch, and raffinose at 1.0% *w/v*), urea, yeast extract, casein, malt extract, proline, and peptone at 0.5%.	Maximal production 113.43 ± 5.11 U/g-ds with 75% of moisture content of 75%, 1 × 10^8^ spores/mL, pH 5.0, at 28 °C for 4 days. Tween 20 enhanced the production by 1.19 folds. Glucose was the best carbon.	[10]
*Sarocladium*	*S. strictum*	MCD L-asparagine	pH 6.8, the incubation period was 2–3 days, carbon sources were D-glucose, starch and molasses, glycerol, ammonium sulphate as a mineral nitrogen source, and soybean powder and yeast extract	1.84-fold increase in enzyme production, $K_{m}$ and V_max_ was 9.74 m/mol and 8.19 mol/ min	[11]

SSF, (solid-state fermentation); SmF, (submerged fermentation); NA, (nutrient agar); MCD, Modified Czapek Dox.

**Table 7:** Examples for the applications of L-Asparaginase as an anti-cancer.

Cancer Type	Preparation	Activity	References
Prostate cancer cell lines lymphoma cancer cells	L-Asparaginase was incorporated into nano biocomposites synthesized using β-cyclodextrin and chitosan.	The mixture exhibited high activity at the concentration of 125 μg/mL and against lymphoma cancer cells (U937) with IC_50_ value at 500 μg/mL of β-cyclodextrin-Asparaginase nanobiocomposite.	[6]
Cervical and brain cancer cell lines	The enzyme was immobilized onto a nanobiocomposite consisting of β-cyclodextrin and Gelatin.	The anticancer activity was 42.13% at 500 μg/mL and 48.60% at 62.5 μg/mL respectively.	[6]
K562 and HL60 cancer cell lines lymphoblastic leukemia	A crude enzyme was mixed with cell viability and then incubated for 24 h at 37 °C inside a CO_2_ incubator, thereafter, ten μL of 10% MTT (3-(4,5-Dimethylthiazol-2-yl)-2,5-diphenyltetrazolium bromide) was added and incubated for 3 h, 100 μL of DMSO was added to the mixture, the absorbance was measured at A_570_ nm.	The toxicity of L-asparaginase against K562 and HL60 cancer cell lines and L6 as normal cells was determined with IC_50_ values were calculated as 0.4 and 0.5 IU/mL for K562 and HL60 respectively	[11]
Childhood leukemia	L-Asparaginase with a dose of ≥ 6000 IU/sq m three times weekly.	L-Asparaginase was effective in re-inducing remissions at 9.5% for 300 IU/sq m; 35.1% for 3000 IU/sq m; 53.5% for 6000 IU/sq m; and 62.5% for 12,000 IU/sq m.	[33]
Acute lymphoblastic leukemia	L-Asparaginase in doses from 10 to 1000 international units/kg body weight per day for 2 to 20 days.	66% response rate for acute lymphoblastic leukemia and an approximately 12% response rate for nonlymphocytic leukemia.	[30]

## Data Availability

Not applicable.

## References

[1] Tran P.N., Yen M.-R., Chiang C.-Y., Lin H.-C., Chen P.-Y. (2019). Detecting and prioritizing biosynthetic gene clusters for bioactive compounds in bacteria and fungi. Appl. Microbiol. Biotechnol..

[2] Deshmukh S.K., Prakash V., Ranjan N. (2018). Marine Fungi: A Source of Potential Anticancer Compounds. Front. Microbiol..

[3] Kumar A., Sørensen J.L., Hansen F.T., Arvas M., Syed M.F., Hassan L., Benz J.P., Record E., Henrissat B., Pöggeler S. (2018). Genome Sequencing and analyses of Two Marine Fungi from the North Sea Unraveled a Plethora of Novel Biosynthetic Gene Clusters. Sci. Rep..

[4] Vala A.K., Sachaniya B., Dudhagara D., Panseriya H.Z., Gosai H., Rawal R., Dave B.P. (2018). Characterization of L-asparaginase from marine-derived Aspergillus niger AKV-MKBU, its antiproliferative activity and bench scale production using industrial waste. Int. J. Biol. Macromol..

[5] Doriya K., Kumar D.S. (2018). Solid state fermentation of mixed substrate for l-asparaginase production using tray and in-house designed rotary bioreactor. Biochem. Eng. J..

[6] Paul V., Tiwary B.N. (2020). An investigation on the acrylamide mitigation potential of l-asparaginase from Aspergillus terreus BV-C strain. Biocatal. Agric. Biotechnol..

[7] El-Gendy M.M.A.A., Awad M.F., El-Shenawy F.S., El-Bondkly A.M.A. (2021). Production, purification, characterization, antioxidant and antiproliferative activities of extracellular L-asparaginase produced by *Fusarium equiseti* AHMF4. Saudi J. Biol. Sci..

[8] Krishnapura P.R., Belur P.D. (2016). Partial purification and characterization of L-asparaginase from an endophytic *Talaromyces pinophilus* isolated from the rhizomes of Curcuma amada. J. Mol. Catal. B Enzym..

[9] Baskar G., Sree N.S. (2020). Synthesis, characterization and anticancer activity of β-cyclodextrin-Asparaginase nanobi-ocomposite on prostate and lymphoma cancer cells. J. Drug Deliv. Sci. Technol..

[10] Jenila A.V., Gnanadoss J.J. (2018). Formulation of A Suitable Medium and its Optimization for Maximizing L-Asparaginase Production from Endophytic Fungi Fusarium sp. LCJ273. Biosci. Biotechnol. Res. Asia.

[11] Fan B., Dewapriya P., Li F., Grauso L., Blümel M., Mangoni A., Tasdemir D. (2020). Pyrenosetin D, a New Pentacyclic Decalinoyltetramic Acid Derivative from the Algicolous Fungus Pyrenochaetopsis sp. FVE-087. Mar. Drugs.

[12] Ashok A., Doriya K., Rao J.V., Qureshi A., Tiwari A.K., Kumar D.S. (2019). Microbes Producing L-Asparaginase free of Glutaminase and Urease isolated from Extreme Locations of Antarctic Soil and Moss. Sci. Rep..

[13] Elshafei A.M., El-Ghonemy D.H. (2015). Screening and media optimization for enhancing L-asparaginase production, an anticancer agent, from different filamentous fungi in solid state fermentation. Biotechnol. J. Int..

[14] Golbabaie A., Nouri H., Moghimi H., Khaleghian A. (2020). L-asparaginase production and enhancement by Sarocladium strictum: In vitro evaluation of anti-cancerous properties. J. Appl. Microbiol..

[15] Nguyen V.-T., Lee J.S., Qian Z.-J., Li Y.-X., Kim K.-N., Heo S.-J., Jeon Y.-J., Park W.S., Choi I.-W., Je J.-Y. (2013). Gliotoxin Isolated from Marine Fungus *Aspergillus* Sp. Induces Apoptosis of Human Cervical Cancer and Chondrosarcoma Cells. Mar. Drugs.

[16] Qi C., Gao W., Guan D., Wang J., Liu M., Chen C., Zhu H., Zhou Y., Lai Y., Hu Z. (2018). Butenolides from a marine-derived fungus Aspergillus terreus with antitumor activities against pancreatic ductal adenocarcinoma cells. Bioorganic Med. Chem..

[17] Malhão F., Ramos A.A., Buttachon S., Dethoup T., Kijjoa A., Rocha E. (2019). Cytotoxic and Antiproliferative Effects of Preussin, a Hydroxypyrrolidine Derivative from the Marine Sponge-Associated Fungus Aspergillus candidus KUFA 0062, in a Panel of Breast Cancer Cell Lines and Using 2D and 3D Cultures. Mar. Drugs.

[18] Chen J.-J., Wang S.-W., Chiang Y.-R., Pang K.-L., Kuo Y.-H., Shih T.-Y., Lee T.-H. (2020). Highly Oxygenated Constituents from a Marine Alga-Derived Fungus Aspergillus giganteus NTU967. Mar. Drugs.

[19] El-Hady F.K.A., Shaker K.H., Souleman A.M.A., Fayad W., Abdel-Aziz M.S., Hamed A.A., Iodice C., Tommonaro G. (2017). Comparative Correlation Between Chemical Composition and Cytotoxic Potential of the Coral-Associated Fungus Aspergillus sp. 2C1-EGY Against Human Colon Cancer Cells. Curr. Microbiol..

[20] Wijesekara I., Zhang C., Van Ta Q., Vo T.-S., Li Y.-X., Kim S.-K. (2014). Physcion from marine-derived fungus Microsporum sp. induces apoptosis in human cervical carcinoma HeLa cells. Microbiol. Res..

[21] Ali T., Inagaki M., Chai H.-B., Wieboldt T., Rapplye C., Rakotondraibe L.H. (2017). Halogenated Compounds from Directed Fermentation of Penicillium concentricum, an Endophytic Fungus of the Liverwort Trichocolea tomentella. J. Nat. Prod..

[22] Jones E.G., Pang K.-L., Abdel-Wahab M.A., Scholz B., Hyde K.D., Boekhout T., Ebel R., Rateb M.E., Henderson L., Sakayaroj J. (2019). An online resource for marine fungi. Fungal Diversity.

[23] Brereton P., Kitchenham B.A., Budgen D., Turner M., Khalil M. (2007). Lessons from applying the systematic literature review process within the software engineering domain. J. Syst. Softw..

[24] Liberati A., Altman D.G., Tetzlaff J., Mulrow C., Gøtzsche P.C., Ioannidis J.P.A., Clarke M., Devereaux P.J., Kleijnen J., Moher D. (2009). The PRISMA statement for reporting systematic reviews and meta-analyses of studies that evaluate health care interventions: Explanation and elaboration. J. Clin. Epidemiol..

[25] Amend A., Burgaud G., Cunliffe M., Edgcomb V.P., Ettinger C.L., Gutiérrez M.H., Heitman J., Hom E.F.Y., Ianiri G., Jones A.C. (2019). Fungi in the Marine Environment: Open Questions and Unsolved Problems. mBio.

[26] Shin H.J. (2020). Natural Products from Marine Fungi. Mar. Drugs.

[27] Zin S.R.M., Kassim N.M., Alshawsh M.A., Hashim N.E., Mohamed Z. (2017). Biological activities of Anastatica hierochuntica L.: A systematic review. Biomed. Pharmacother..

[28] Hu Y., Chen J., Hu G., Yu J., Zhu X., Lin Y., Chen S., Yuan J. (2015). Statistical Research on the Bioactivity of New Marine Natural Products Discovered during the 28 Years from 1985 to 2012. Mar. Drugs.

[29] Petersen L.-E., Kellermann M.Y., Schupp P.J. (2019). Secondary Metabolites of Marine Microbes: From Natural Products Chemistry to Chemical Ecology. Youmares 9—The Oceans: Our Research, Our Future.

[30] Hasan S., Ansari M.I., Ahmad A., Mishra M. (2015). Major bioactive metabolites from marine fungi: A Review. Bioinformation.

[31] Oda T., Namikoshi M., Akano K., Kobayashi H., Honma Y., Kasahara T. (2005). Verrucarin A Inhibition of MAP Kinase Activation in a PMA-stimulated Promyelocytic Leukemia Cell Line. Mar. Drugs.

[32] Zabielska J., Sledzinski T., Stelmanska E. (2019). Acyl-Coenzyme A: Cholesterol Acyltransferase Inhibition in Cancer Treatment. Anticancer. Res..

[33] Grinde M.T., Hilmarsdottir B., Tunset H.M., Henriksen I.M., Kim J., Haugen M.H., Rye M.B., Mælandsmo G.M., Moestue S.A. (2019). Glutamine to proline conversion is associated with response to glutaminase inhibition in breast cancer. Breast Cancer Res..

[34] Li Y.L., Li Q.X., Liu R.J., Shen X.Q. (2018). Chinese medicine Amygdalin and β-glucosidase combined with antibody en-zymatic prodrug system as a feasible antitumor therapy. Chin. J. Integr. Med..

[35] Feng Y., Xiong Y., Qiao T., Li X., Jia L., Han Y. (2018). Lactate dehydrogenase A: A key player in carcinogenesis and potential target in cancer therapy. Cancer Med..

[36] Guest T.C., Rashid S. (2016). Anticancer laccases: A review. J. Clin. Exp. Oncol. 5.

[37] Su Y.-C., Cheng T.-C., Leu Y.-L., Roffler S.R., Wang J.-Y., Chuang C.-H., Kao C.-H., Chen K.-C., Wang H.-E., Cheng T.-L. (2014). PET Imaging of β-Glucuronidase Activity by an Activity-Based 124I-Trapping Probe for the Personalized Glucuronide Prodrug Targeted Therapy. Mol. Cancer Ther..

[38] Fedrowitz M., Hass R., Bertram C., Löscher W. (2011). Salivary α-amylase exhibits antiproliferative effects in primary cell cultures of rat mammary epithelial cells and human breast cancer cells. J. Exp. Clin. Cancer Res..

[39] Mook O.R., Frederiks W.M., Van Noorden C.J. (2004). The role of gelatinases in colorectal cancer progression and metastasis. Biochim. Biophys. Acta.

[40] Saber-Moghaddam N., Nomani H., Sahebkar A., Johnston T.P., Mohammadpour A.H. (2019). The change of immunosuppressive regimen from calcineurin inhibitors to mammalian target of rapamycin (mTOR) inhibitors and its effect on malignancy following heart transplantation. Int Immunopharmacol..

[41] Liu Y.Q., Zhou T., Zhao Y.Y., Chen L., Gong W.M., Xia W.Q., Ying G.M., Zheng H.Q., Zhang Q.Q. (2015). Antitumor effects and related mechanisms of penicitrinine A, a novel alkaloid with a unique spiro skeleton from the marine fungus Penicillium citrinum. Mar. Drugs.

[42] Law B.K. (2005). Rapamycin: An anti-cancer immunosuppressant?. Crit. Rev. Oncol..

[43] Blagosklonny M.V. (2019). Rapamycin for longevity: Opinion article. Aging.

[44] Dosio F., Arpicco S., Stella B., Fattal E. (2016). Hyaluronic acid for anticancer drug and nucleic acid delivery. Adv. Drug Deliv. Rev..

[45] Wakharde A.A., Awad A.H., Bhagat A., Karuppayil S.M. (2018). Synergistic Activation of Doxorubicin against Cancer: A Review. Am. J. Clin. Microbiol. Antimicrob..

[46] Falini B., Brunetti L., Martelli M.P. (2015). Dactinomycin in NPM1-Mutated Acute Myeloid Leukemia. N. Engl. J. Med..

[47] Abotaleb M., Samuel S.M., Varghese E., Varghese S., Kubatka P., Líšková A., Büsselberg D. (2018). Flavonoids in Cancer and Apoptosis. Cancers.

[48] Shao R.-G., Zhen Y.-S. (2008). Enediyne anticancer antibiotic lidamycin: Chemistry, biology and pharmacology. Ant Cancer Agents Med. Chem..

[49] Gederaas O.A., Søgaard C., Viset T., Bachke S., Bruheim P., Arum C.-J., Otterlei M. (2014). Increased Anticancer Efficacy of Intravesical Mitomycin C Therapy when Combined with a PCNA Targeting Peptide. Transl. Oncol..

[50] Dasari S., Tchounwou P.B. (2014). Cisplatin in cancer therapy: Molecular mechanisms of action. Eur. J. Pharmacol..

[51] Yu Z., Yan B., Gao L., Dong C., Zhong J., D’Ortenzio M., Nguyen B., Lee S.S., Hu X., Liang F. (2016). Targeted Delivery of Bleomycin: A Comprehensive Anticancer Review. Curr. Cancer Drug Targets.

[52] Huang C., Jin H., Song B., Zhu X., Zhao H., Cai J., Lu Y., Chen B., Lin Y. (2012). The cytotoxicity and anticancer mechanisms of alterporriol L, a marine bianthraquinone, against MCF-7 human breast cancer cells. Appl. Microbiol. Biotechnol..

[53] Pang X., Lin X., Wang P., Zhou X., Yang B., Wang J., Liu Y. (2018). Perylenequione Derivatives with Anticancer Activities Isolated from the Marine Sponge-Derived Fungus, Alternaria sp. SCSIO41014. Mar. Drugs.

[54] Ramos A.A., Castro-Carvalho B., Prata-Sena M., Malhão F., Buttachon S., Dethoup T., Kijjoa A., Rocha E. (2020). Can marine-derived fungus Neosartorya siamensis KUFA 0017 extract and its secondary metabolites enhance antitumor activity of doxorubicin? An in vitro survey unveils interactions against lung cancer cells. Environ. Toxicol..

[55] Mathan S., Smith A.A., Kumaran J., Prakash S. (2011). Anticancer and Antimicrobial Activity of Aspergillus protuberus SP1 Isolated from Marine Sediments of South Indian Coast. Chin. J. Nat. Med..

[56] Ding Y.-S., Kim W.-S., Park S.J., Kim S.-K. (2018). Apoptotic effect of physcion isolated from marine fungus Microsporum sp. in PC3 human prostate cancer cells. Fish. Aquat. Sci..

[57] Eamvijarn A., Kijjoa A., Bruyère C., Mathieu V., Manoch L., Lefranc F., Silva A., Kiss R., Herz W. (2012). Secondary Metabolites from a Culture of the Fungus Neosartorya pseudofischeri and Their In Vitro Cytostatic Activity in Human Cancer Cells. Planta Medica.

[58] Dezaire A., Marchand C.H., Vallet M., Ferrand N., Chaouch S., Mouray E., Larsen A.K., Sabbah M., Lemaire S.D., Prado S. (2020). Secondary Metabolites from the Culture of the Marine-derived Fungus Paradendryphiella salina PC 362H and Evaluation of the Anticancer Activity of Its Metabolite Hyalodendrin. Mar. Drugs.

[59] Farha A.K., Hatha A.M. (2019). Bioprospecting potential and secondary metabolite profile of a novel sediment-derived fungus Penicillium sp. ArCSPf from continental slope of Eastern Arabian Sea. Mycology.

[60] Parthasarathy R., Chandrika M., Rao H.Y., Kamalraj S., Jayabaskaran C., Pugazhendhi A. (2020). Molecular profiling of marine endophytic fungi from green algae: Assessment of antibacterial and anticancer activities. Process. Biochem..

[61] Oppong-Danquah E., Passaretti C., Chianese O., Blümel M., Tasdemir D. (2020). Mining the Metabolome and the Agricultural and Pharmaceutical Potential of Sea Foam-Derived Fungi. Mar. Drugs.

[62] Mohamed I.E., Gross H., Pontius A., Kehraus S., Krick A., Kelter G., Maier A., Fiebig H.-H., Koenig G.M. (2010). ChemInform Abstract: Epoxyphomalin A (I) and B (II), Prenylated Polyketides with Potent Cytotoxicity from the Marine-Derived Fungus Phoma sp.. Org. Lett..

[63] Anand B.G., Thomas C.N., Prakash S. (2016). In vitro cytotoxicity and antimicrobial activity of Talaromyces flavus SP5 in-habited in the marine sediment of Southern Coast of India. Chin. J. Nat. Med..

[64] Kebede B., Wrigley S.K., Prashar A., Rahlff J., Wolf M., Reinshagen J., Gribbon P., Imhoff J.F., Silber J., Labes A. (2017). Establishing the Secondary Metabolite Profile of the Marine Fungus: Tolypocladium geodes sp. MF458 and Subsequent Optimisation of Bioactive Secondary Metabolite Production. Mar. Drugs.

[65] Bedaiwy M.Y., Awadalla O.A., Abou-Zeid A.M., Hamada H.T. (2016). Optimal conditions for production of L-asparaginase from Aspergillus tamarii. Egypt. J. Exp. Biol..

[66] Da Rocha W.R.V., Costa-Silva T.A., Agamez-Montalvo G.S., Feitosa V.A., Machado S.E.F., de Souza Lima G.M., Pessoa A., Alves H.S. (2019). Screening and optimizing fermentation production of l-asparaginase by Aspergillus terreus strain S-18 isolated from the Brazilian Caatinga Biome. J. Appl. Microbiol..

[67] Da Cunha K.C., Sutton D.A., Fothergill A.W., Gené J., Cano J., Madrid H., de Hoog S., Crous P.W., Guarro J. (2013). In vitro antifungal susceptibility and molecular identity of 99 clinical isolates of the opportunistic fungal genus Curvular-ia. Diagn. Microbiol. Infect. Dis..

[68] El-Said A.H., Shebany Y.M., Hussein M.A., El-Dawy E.G. (2016). Antimicrobial and L-asparaginase activities of endophytic fungi isolated from Datura innoxia and Hyoscyamus muticus medicinal plants. Eur. J. Biol. Res..

[69] Gonçalves A.B., Maia A.C.F., Rueda J.A., Vanzela A.P.D.F.C. (2016). <b> Fungal production of the anti-leukemic enzyme L-asparaginase: From screening to medium development. Acta Sci. Biol. Sci..

[70] Cardoso S.L., De Freitas M.M., De Souza P.M., Homem-De-Mello M., Silveira D., Fonseca-Bazzo Y.M., Filho E.X., Junior A.P., Magalhães P.O. (2020). Optimization of aqueous two-phase micellar system for partial purification of L-asparaginase from Penicillium sp. grown in wheat bran as agro-industrial residue. Braz. J. Microbiol..

[71] Vieira W.F., Correa H.T., Campos E.S., Sette L.D., Pessoa Jr A., Cardoso V.L., Coutinho Filho U. (2020). A novel multiple reactor system for the long-term production of L-asparaginase by *Penicillium* sp. LAMAI 505. Process Biochem..

